# Simulation of the activation of mining faults and grouting reinforcement under thick loose layer and thin bedrock

**DOI:** 10.1038/s41598-022-21654-x

**Published:** 2022-10-11

**Authors:** Wenquan Zhang, Yu Lei, Jianli Shao, Xunan Wu, Song Li, Chaoqun Ma

**Affiliations:** 1 State Key Laboratory of Strata Intelligent Control and Green Mining Co-founded by Shandong Province and the Ministry of Science and Technology, Qingdao, 266590 China; 2 National Experimental Teaching Demonstration Center for Mining Engineering, Shandong University of Science and Technology, Qingdao, 266590 China

**Keywords:** Civil engineering, Hydrology

## Abstract

We have investigated the activation characteristics of mining faults and the effect of grouting reinforcement under thick loose layer and thin bedrock of the working face and evaluate their impact on the safety of mining at similar working faces. Implementing the geological conditions of the 331 working face of the Yangcun Coal Mine (China) of the Yankuang Energy Group Corporation, we have analyzed mechanically the process of fault activation at first. Subsequently, we have obtained the mechanical criterion of fault slip and the expression of relative strength of the nearby rock mass. Using numerical software we have simulated and analyzed the damage characteristics of different bedrock thicknesses on overlying rocks and faults in the fluid–solid coupling mode. In addition, we have studied the controlling effect of grouting reinforcement on fault activation, which has been verified in the field. The main results of our study show that: 1. The mechanical properties of the rock mass near the fault interface have changed and they are related to the cohesive force of the interface; 2. The water inrush mode of the working face changes under different bedrock thickness, and the thinner the bedrock, the less easily the fault is destroyed 3. The slip of the high-level fault is reduced after the grouting of the fault, the propagation of the fracture zone at the fault is suppressed, the seepage of the aquifer water is prevented, and the safe recovery is realized. The results of our study provide a scientific basis for the secure mining across the fault of the 331 working face of Yangcun Coal Mine. Based on the results of our study the working face can be mined safely from now on and in the future.

## Introduction

Coal is the most important fossil energy source in China. According to the bulletin of the National Bureau of Statistics, China's raw coal production will reach 4.13 billion tons in 2021. In 2021, China's raw coal production and consumption will increase by 5.7% and 4.6%, respectively, and coal consumption will account for 56.0% of the total energy consumption. Coal mine water inrush seriously endanger the safety of workers and the progress of the mine production^1–3^. On July 5, 2012, a fault was induced in the Jialichong Xinjing Coal Mine in Leiyang City (Hunan Province, China) due to the change of the geological conditions during the excavation process, and the surrounding rock of the roadway collapsed, causing a water inrush accident and 16 workers were trapped underground. On March 11, 2013, the Heilongjiang Zhenxing Coal Mine (China) was mined for extra-thick coal seams due to top coal caving in the lower wall of the reverse fault, resulting in the development of water-conducting fracture zones which involved the upper coal seam and the fault zone. The process induced a major water accident at the working face and 18 worker lost their life. The economic loss was 22.81 million yuan. On October 25, 2019, blasting operation at the working face in the Xigu County Coal Mine of Xiangkuang, Changzhi City, Shanxi Province (China), caused the loosing of the fault fracture zone, which finally collapsed under the action of water pressure, leading to an old empty area and causing a large water disaster accident. Four miners lost their life and the economic loss was 9.68 million yuan. The examples of the mining accident demonstrate that coal seam mining may lead to fault activation with mine water inrush as one of the urgent problems that need conclusive solution.

To solve the problem of fault activation during mining operation, numerous studies have been conducted in recent years: Wankui et al.^4^ used the elasticity theory to analyze the relationship between the dip angle of normal faults and their activation, and concluded that normal faults with small dip angles are easier to activate. Used numerical simulation, Yaodong et al.^5^ studied the influence of different mining methods on fault activation and concluded that the impact of footwall mining on faults is more enhanced, and its activation risk is higher. Qingfeng et al.^6^ studied the activation water inrush mechanics model of the water-proof key layer containing water-proof faults, and proposed the water inrush conditions and water inrush principle of the fault mining activation under the combined action of mineral pressure and water pressure. Shucai et al.^7^ analyzed the filling medium. Based on the mechanical properties and seepage properties, they established the swallowtail catastrophe model of the sliding water inrush of the backfill structure. Aiwen et al.^8–12^ used similar material simulation experiments to study the stress change law of the fault under the influence of mining. Shao et al.^13^ established a multi-field coupled seepage characteristic evolution model to study the evolution process of water inrush inside the fault and the influence of water pressure, initial effective porosity, and initial permeability on water flow. Peisen et al.^14,15^ used numerical software to simulate the dip angle of the fault, the impact of drop, and broken zone width change on the stress and slip of the fault plane in the solid–liquid coupling mode. Using numerical simulations, Li et al.^16–21^ analyzed the coupled evolution process of stress field, displacement field and seepage field, and summarized the precursory information characteristics of fault activation.

The bond strength of the fault plane and the influence of the fault grouting reinforcement on the fault slip were not considered in the previous studies. The 331 working face of Yangcun Coal Mine of Yankuang Energy Group Co.Ltd. is part of the mining area under thin bedrock and thick loose aquifer. The normal fault of Quince Tree No. 2 in the working face crosses the working face obliquely from the upper left end of the incision. The working face must be mined across the fault, which is very risky. Therefore, we have developed a plan for grouting and strengthening of the fault prior to the mining. To reduce the probability of water gushing and sand inrush accidents caused by fault activation, we studied the failure characteristics of faults and the effect of grouting reinforcement under different bedrock thickness conditions based on the mechanism of fault activation mechanics and the analysis of the bonding force of the fault zone. Based on the degree of fault activation control we can evaluate the mining safety of the working face, providing a reliable scientific basis for the safe production of the working face.

## Mechanical analysis of fault activation mechanism

**Figure 1:**
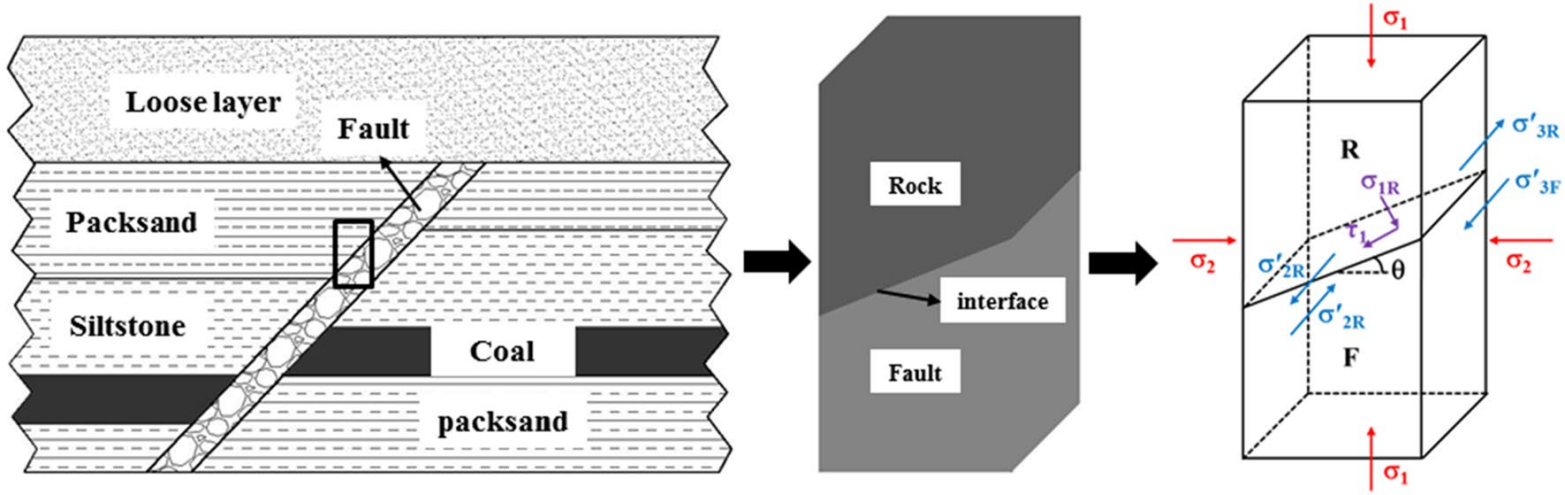
Force diagram of mechanical model. (R: Rock mass; F: Fault zone; *σ*_1_: Axial force on the structure; *θ*: Fault dip angle; *σ*_1*R*_ and *τ*_1_: Two component forces of *σ*_1_ on the contact surface, *σ′*_2*R*_ and *σ′*_2*F*_: Rock mass and binding force of the fault zone in the second direction at the contact surface; *σ′*_3*R*_ and *σ′*_3*F*_: Binding force of the rock mass and the fault zone at the contact surface in the third direction).

After excavation of the coal seam, the fault is activated through the influence of mining. To study the mechanical behavior of the rock mass near the fault and the conditions for slipping, we simplified the actual fault as a rock-fault zone structure with a contact surface (see Fig. 1) mechanical analysis.

According to the Mohr–Coulomb criterion, the residual shear force of the contact surface slipping is obtained as:

1 $$ \left\{ {\begin{array}{*{20}l} {\tau e = \tau 1 - cj = \sigma_{1} \sin \theta + \sigma_{2} \cos \theta - cj} \hfill & {\tau 1 > cj} \hfill \\ {\tau e = 0} \hfill & {\tau 1 \le cj} \hfill \\ \end{array} } \right. $$

with *τ*_*e*_ as the residual shear stress at the contact surface, *τ*_1_ as the shear stress on the contact surface, and *c*_*j*_ as the bonding force of the contact surface.

According to the generalized Hooke's law and Moore's Coulomb strength theory, the axial ultimate strengths of the rock mass near the contact surface are obtained as:

2 $$ \left\{ {\begin{array}{*{20}l} {\sigma ^{\prime}_{1Rj} = \frac{{R_{CR} - a_{R} cj}}{{1 + a_{R} (f_{RF} - \tan \theta )}}} \hfill & {\tau e > 0} \hfill \\ {\sigma ^{\prime}_{1Rj} = \frac{{R_{CR} }}{{1 + a_{R} f_{RF} }}} \hfill & {\tau e = 0} \hfill \\ \end{array} } \right. $$

3 $$ \left\{ {\begin{array}{*{20}l} {\sigma ^{\prime}_{1Fj} = \frac{{R_{CF} + a_{F} cj}}{{1 + a_{F} (\tan \theta - f_{RF} )}}} \hfill & {\tau e > 0} \hfill \\ {\sigma ^{\prime}_{1Fj} = \frac{{R_{CF} }}{{1 - a_{F} f_{RF} }}} \hfill & {\tau e = 0} \hfill \\ \end{array} } \right. $$

in:

4 $$ \left\{ {\begin{array}{*{20}l} {a_{R} = \frac{{1 + \sin \varphi_{R} }}{{1 - \sin \varphi_{R} }}} \hfill \\ {a_{F} = \frac{{1 + \sin \varphi_{F} }}{{1 - \sin \varphi_{F} }}} \hfill \\ \end{array} } \right. $$

with *Rc* as the uniaxial compressive strength of the rock mass; *f*_*RF*_ as the friction coefficient at the contact surface; and *φ* as the internal friction angle of the rock mass.

If the residual shear force τe is > 0, the structure body slides along the contact surface according to Eq. (1), and the greater the stress (*σ*_1_) of the fractured rock mass, the easier the structure body slides along the contact surface. In the peak area of supporting pressure, i.e., in front of the coal wall of the coal mining face, the fault activation is more likely to occur. Equations (2) and (3) indicate that, for fixed values of other parameters, the greater the bonding force of the contact surface, the greater the degree of strengthening of the fault zone, and the lower the strength required for the rock at the contact surface. On the contrary, the smaller the bonding force of the contact surface, the lower the degree of strengthening of the fault zone, and the greater the strength required for the rock at the contact surface, which is reflected in good coordination and consistency. This shows that if the fault zone at the peak area of supporting pressure in front of the coal wall of the coal mining face is not activated, it is necessary to increase the bonding force between the fault zone and the contact surface of the rock formation (which can be achieved by grouting) or to improve the strength of the rock formation. The peak area of supporting pressure in front of the coal wall of the underground coal mining face is bound to appear, and the seepage of water is very likely to occur in the near loose aquifer (even the weak aquifer). The water-bearing situation in the fault zone is also common. However, the previous analytical equation does not consider the influence of confined water and fluid–solid coupling. Moreover, it is difficult to accurately describe the activation characteristics of fault zones and the effect of grouting reinforcement of fault zones in the dynamic process of production for the three-dimensional underground space. Therefore, we use the cross-fault mining under the thin bedrock and thick loose layer of the 331 working face of the Yangcun Coal Mine of Yankuang Energy Group Corporation as a case study. Based on the mechanical analysis, the FLAC3D numerical simulation software is used to conduct various methods to model the slip failure of the fault. The results will improve the safety and feasibility of cross-fault mining under the thin bedrock and thick loose aquifer of the 331 working face.

## Engineering background

### Coal seam and roof and floor characteristics

**Figure 2:**
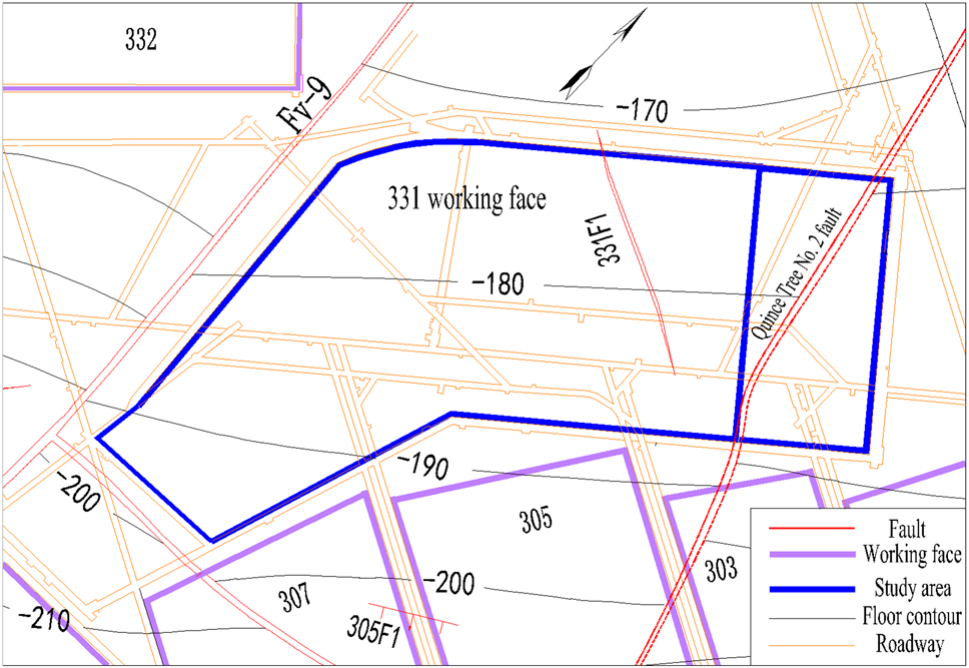
331 working face of Yangcun Coal Mine.

The research area studied in our project is the designed mining area of the 331 working face of Yangcun Coal Mine. The length of the working face is 452–610 m, the width of the cut hole is about 182 m, the width of the stop line is about 100 m, and the area is about 89,309 m^2^ (Fig. 2). The buried depth of the 3 coal seam is 215.8 m, the dip angle of the coal seam is 1° to 6°, with an average of 4°, and the thickness of the coal seam is 7.75–9.20 m, with an average of 8.20 m. The bedrock thickness is 15.9–49.3 m. Most of the area 3 coal seams are less than 40 m away from the Quaternary loose layer aquifer. The Quince Tree No. 2 normal fault (with a fall of 6 m, an inclination of 50°, and width of 3 m) cross-cuts the 331 working face (Fig. 2), posing a serious threat to safe mining of the working face.

The direct roof of the 3 coal seam is siltstone, ranging from 3.22 to 6.33 m, with an average of 4.36 m. The basic roof is dominated by interbeds of medium to fine sandstone, with a thickness of 10.69–17.06 m, with an average of 12.67 m. The thickness is uniform, but abundant fissures are developed. False tops occur, mostly mudstone, aluminous mudstone, or carbonaceous mudstone. The direct bottom is sandy mudstone with a thickness of 0.00–2.40 m and an average of 1.20 m. The basic bottom is fine sandstone with a thickness of 5.00–5.05 m and an average of 5.02 m, which is relatively stable.

### Water richness analysis

Results of the observation of the water level in an observation hole, indicate that the lower group of the Quaternary, i.e., the "bottom containment", is the main water-filled aquifer on the roof of the 331 working face, which has no necessary hydraulic connection with other aquifers. The unit water inflow q is 0.002 3–0.074 274 L/(s∙m), demonstrating that the water richness is weak, and the replenishment is not smooth. It is a weak water rich aquifer, and the current water level shows only minor changes. The cohesive soil layer at the bottom of the Quaternary system is a hard, semi-consolidated and dense water-saturated soil with low compressibility, poor permeability, and good expansion. The soil layer is an effective cohesive soil water barrier with a variable thickness of 0.00–5.74 m.

## Numerical simulation

### Construction of a numerical model

**Figure 3:**
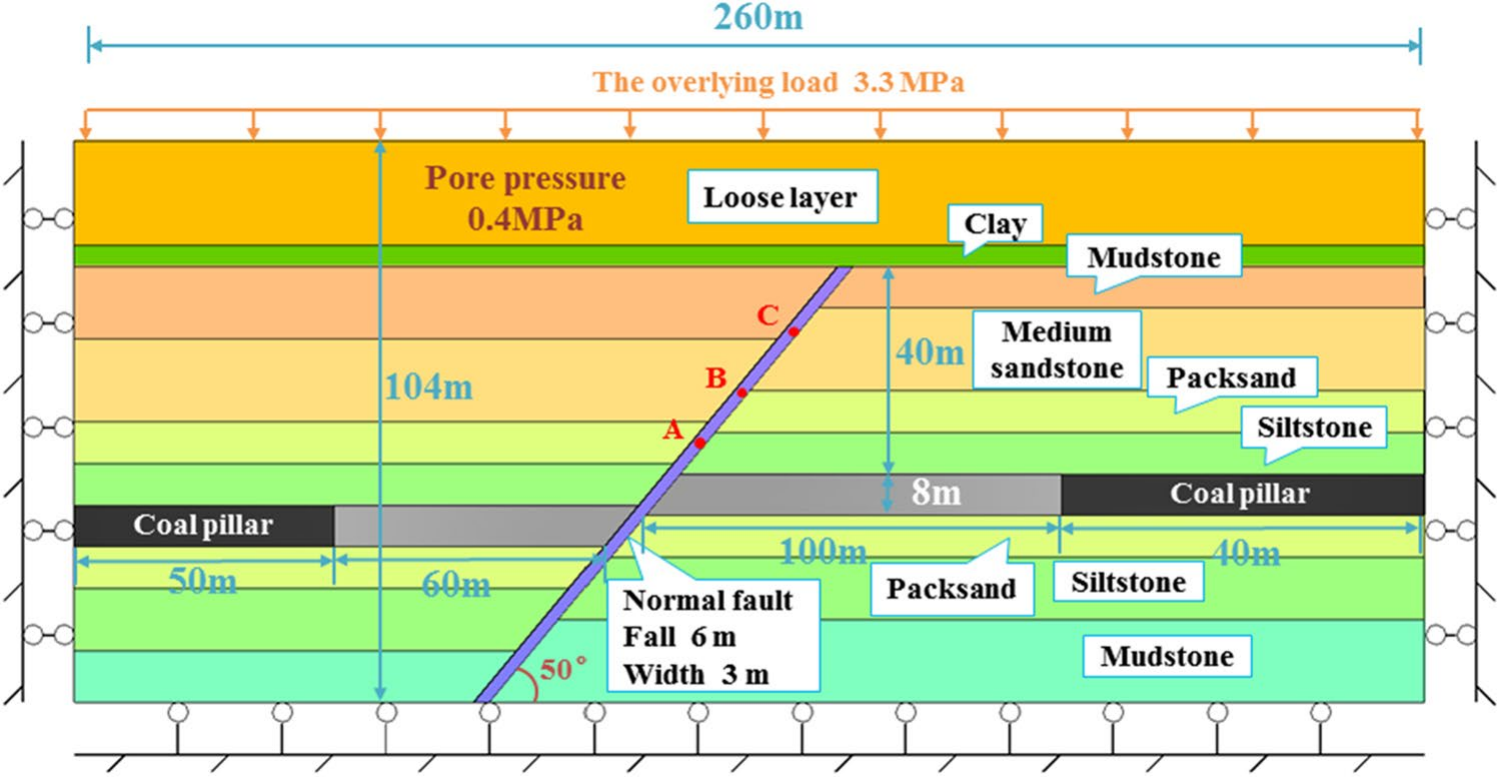
Schematic model of the geological situation at the 331 working face.

At first we established the FLAC3D numerical simulation model was first to study the risk of water gushing and sand inrush caused by the activation of faults in the 331 working face of Yangcun Coal Mine during the mining process. Considering the geological conditions of the 331 working face, the size of the model is 260 m × 50 m × 104 m (the model heights are 84 m and 94 m when the bedrock thickness is 20 m and 30 m, respectively). Horizontal constraints were applied to the front, rear, and left and right boundaries. The bottom boundary was fixed. The the upper part is a free boundary and a load of 3.3 MPa is applied to replace the overlying gravity. The water pressure of the loose layer is set to 0.4 MPa. The Molecular-Coulomb criterion is used for the mechanical calculation, and an isotropic, uniform, and equivalent continuum model is used for fluid calculation. The left and right sides of the model are left to protect coal pillars. The coal thickness is 8 m and the coal mining process is fully mechanical (along the coal seam floor). The mining height is 3 m and the mining method is the forward type, gradually approaching the fault with an advance of 10 m each time towards the fault. The monitoring points A, B, and C are arranged in the belt, 5 m, 15 m, and 25 m away from the upper part of the coal seam, respectively, as shown in Fig. 3.

According to the results of the on-site geological investigation and relevant rock mechanics experiments, and considering the size effect of the mine rock mass, suited calculation parameters were selected for the numerical calculation that are summarized in Table 1. To improve the accuracy of the simulation, the seepage parameters of the rock mass after plastic failure were modified with the *fish* language^22^.

**Table 1:** Physical and mechanical parameters of the rock sequence at the 331 working face.

Lithology	Bulk modulus (Gpa)	Shear modulus (Gpa)	Cohesion (MPa)	Friction Angle (°)	Tensile strength (MPa)	Permeability coefficient (cm/s)	Porosity (n)	Density (kg m^−3^)
loose layer	2.1	1.7	0.11	10	0.5	4e−3	0.38	2100
clay	0.28	0.09	0.85	25	0.35	9e−7	0.35	1960
mudstone	1.2	1.23	1.6	26	0.6	2.5e−5	0.25	2450
medium sandstone	13.4	8.2	4.8	33	1.73	8.32e−5	0.28	2550
fine sandstone	10.8	7.6	4.5	35	2.0	3.7e−5	0.30	2650
siltstone	5	3.8	2.55	35	1.74	4.1e−5	0.26	2460
coal	4.8	2.4	2.2	28	1.9	4.62e−4	0.35	1400
fault	0.27	0.21	0.4	26	0.1	1.6e−3	0.46	1800

### Overburden and fault failure under different bedrock thickness conditions

**Figure 4:**
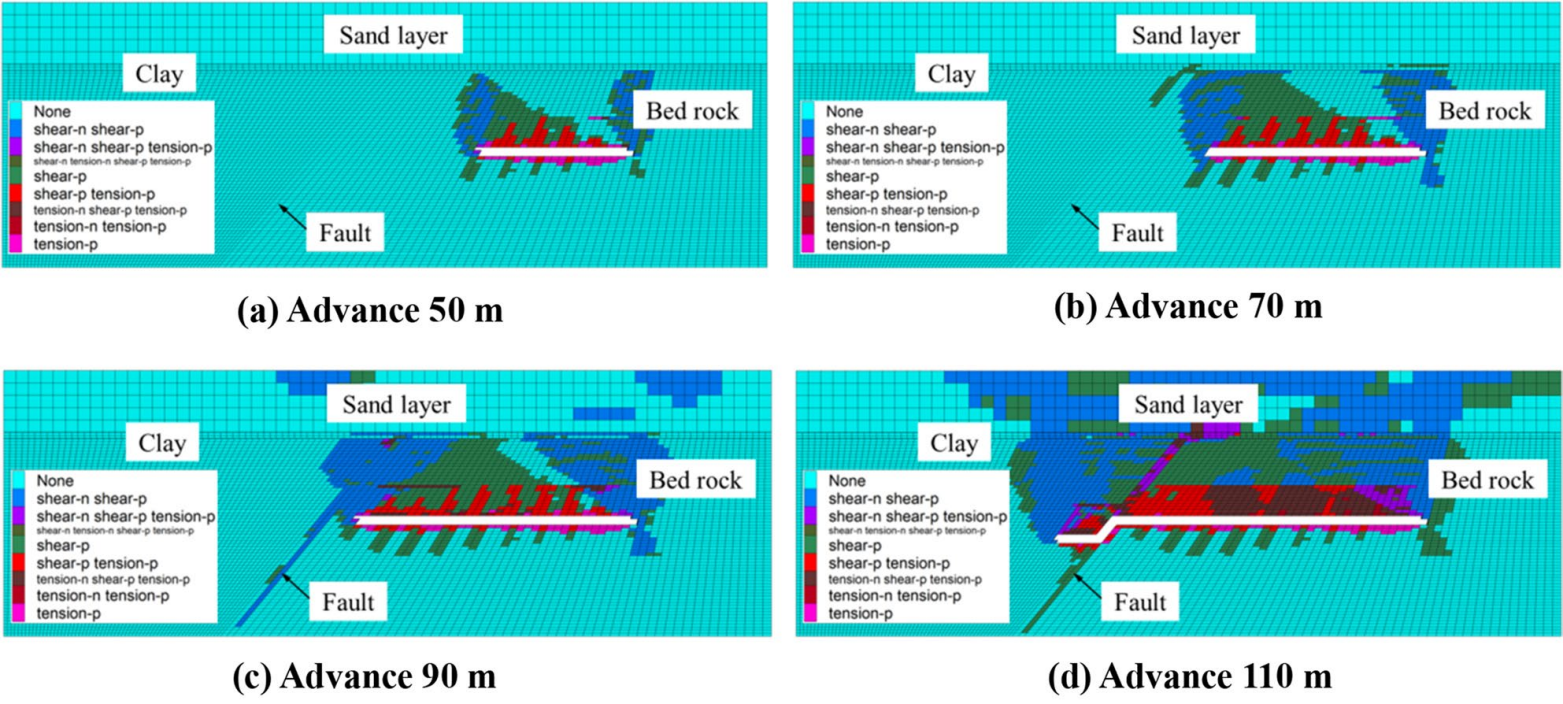
Plastic failure diagram of the model for a bedrock thickness of 20 m.

Figure 4 shows the plastic failure diagram of the model in the process of working face propulsion for a bedrock thickness of 20 m (shear-n means that the element is currently being shear damaged, shear-p means that the element has been shear damaged before; tension-n means that the element is currently being tension damaged, tension-p means that the element has been tension damaged before). According to the “Regulations for coal pillar retention and coal press mining for building water bodies, railways and main shafts”, the mining height is 3 m below the condition of a medium-hard rock. The height of the caving zone is 6.9–11.3 m. The development height of the fracture zone is 30.1–41.3 m. It is evident that the thickness of the bedrock is not satisfied with the development height of the fracture zone. According to the size of the model and the form of the rock mass failure, the height of the caving zone and the fracture zone can be adjusted^23^. For a progress of 50 m of the working face, the caving zone develops to 10 m and strops rising. Moreover, the fracture zone has developed to the top of the bedrock. For a advance of 70 m of the working face, the height of the fracture zone does not change, because the clay layer can temporarily inhibit the development of fractures until it is completely destroyed^24^. At this time, the top of the fault zone starts to be affected by plastic failure, and becomes connected to the fracture zone. The clay layer on-top shows through-type fractures, which connect the overlying loose aquifer to form a water-conducting channel. When the working face advances up to 90 m, a through-type fracture appeared in the clay layer overlying the opening, connecting the loose aquifer to form a water-conducting channel, and the fault and the rock mass of the upper and lower walls became affected by large-scale damage. After 110 m advance of the working face, it had passed through the fault zone for 10 m at this time. The rock mass above the mining face has been completely destroyed, the water-blocking ability of the clay layer has been completely lost, and the fault zone has been rotated due to the mining of the working face^25^. As a consequence, the original shear failure has been transformed into tensile failure.

**Figure 5:**
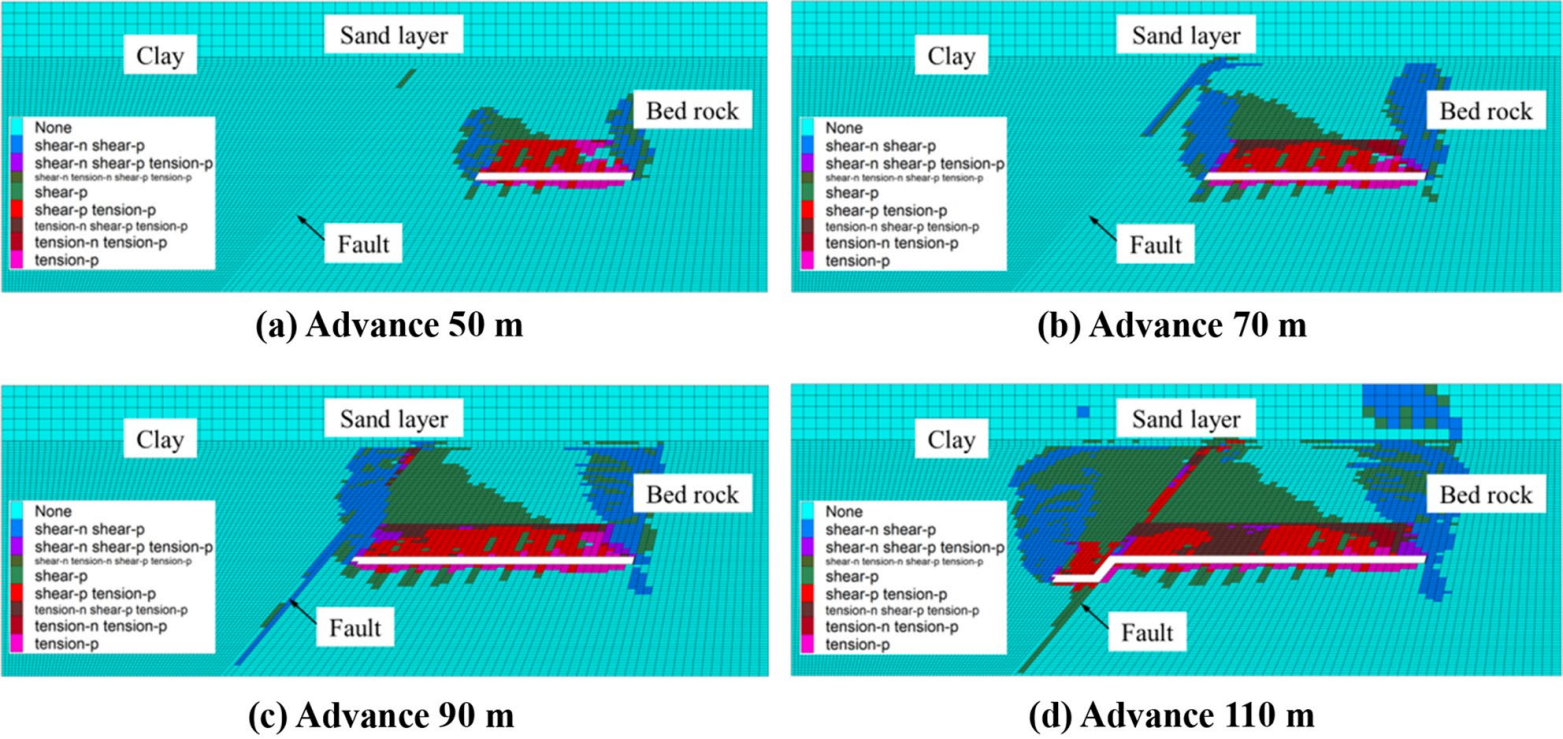
Plastic failure diagram of the model for a bedrock thickness of 30 m.

Figure 5 shows the plastic failure diagram of the model in the process of working face propulsion for a bedrock thickness of 30 m. When the working face is advanced by 50 m, the caving zone develops to 10 m, and the fracture zone develops to 26 m. At this time, the fault becomes affected by slight shear damage. When the working face is advanced to 70 m, the fracture zone develops to the top of the bedrock and becomes connected with the fault zone failure area. The clay layer overlying the fault is destroyed and connected with the loose aquifer. When the working face is advanced by 90 m, the height of the fracture zone remains unchanged and the clay layer above the incision hole remains unmodified. Partially damaged but not penetrated, it still maintains a good water blocking capacity and the clay layer above the fault is damaged. When the working face advances toward 110 m, the damage range of the clay layer above the incision hole is enlarged and the damage of the clay layer on the upper wall of the fault is low.

**Figure 6:**
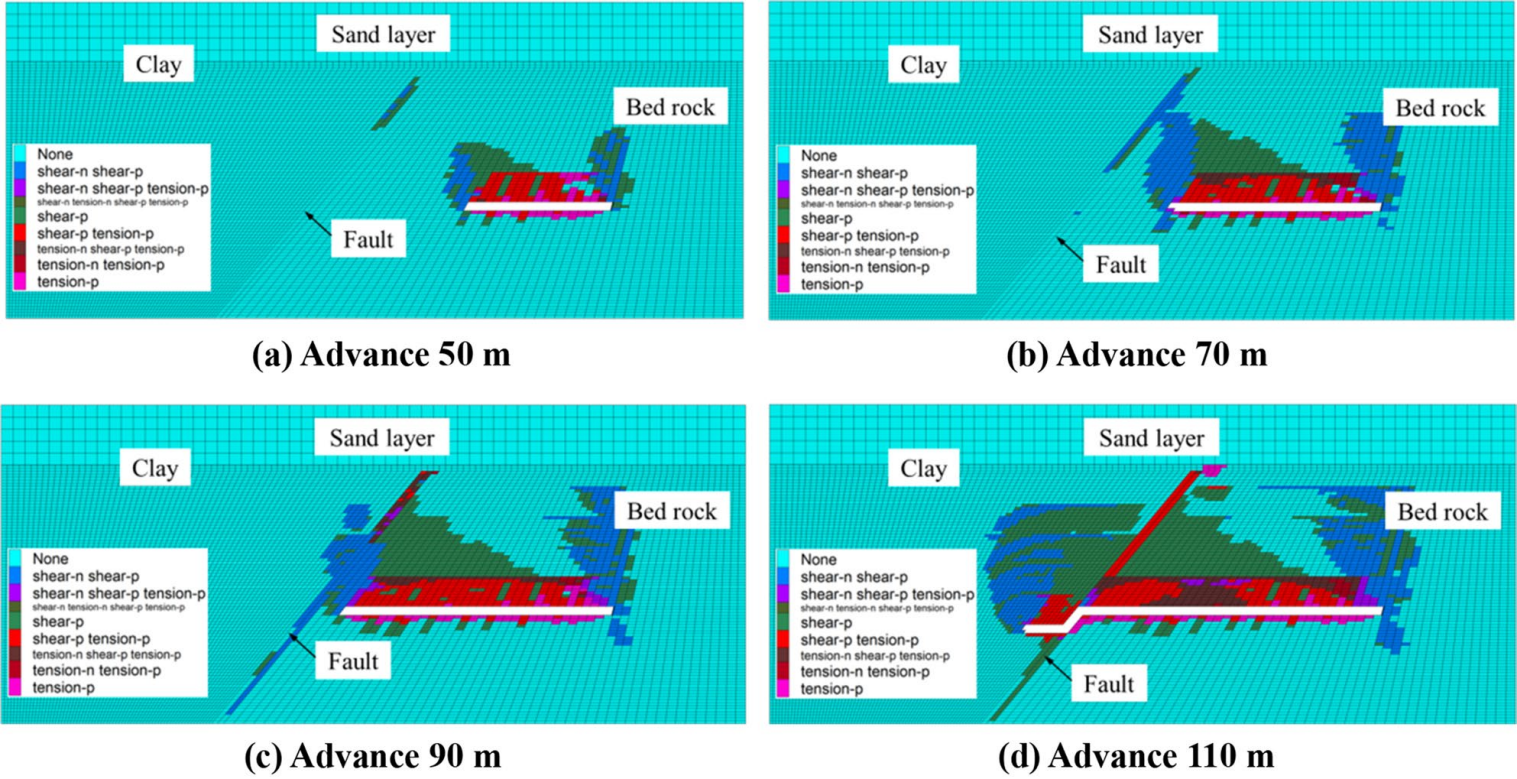
Plastic failure diagram of the model for a bedrock thickness of 40 m.

Figure 6 shows the plastic failure diagram of the model in the process of working face propulsion for a bedrock thickness of 40 m. When the working face is advanced by 50 m, the fracture zone develops to 26 m and the fault zone becomes affected by plastic failure. When the working face is advanced by 70 m, the fracture zone interacts with the fault plastic zone. When the working face is advanced by 90 m, the fracture zone develops to 40 m. Due to the "barrier" effect of the fault^26^, the area of the plastic zone on the fault hanging wall is relatively small. When the working face is advanced by 110 m, the height of the fracture zone remains unchanged, but a through-type fracture appears in the clay layer above the fault, linking the water-bearing sand layer with the fault failure area, thus forming a water channel.

**Figure 7:**
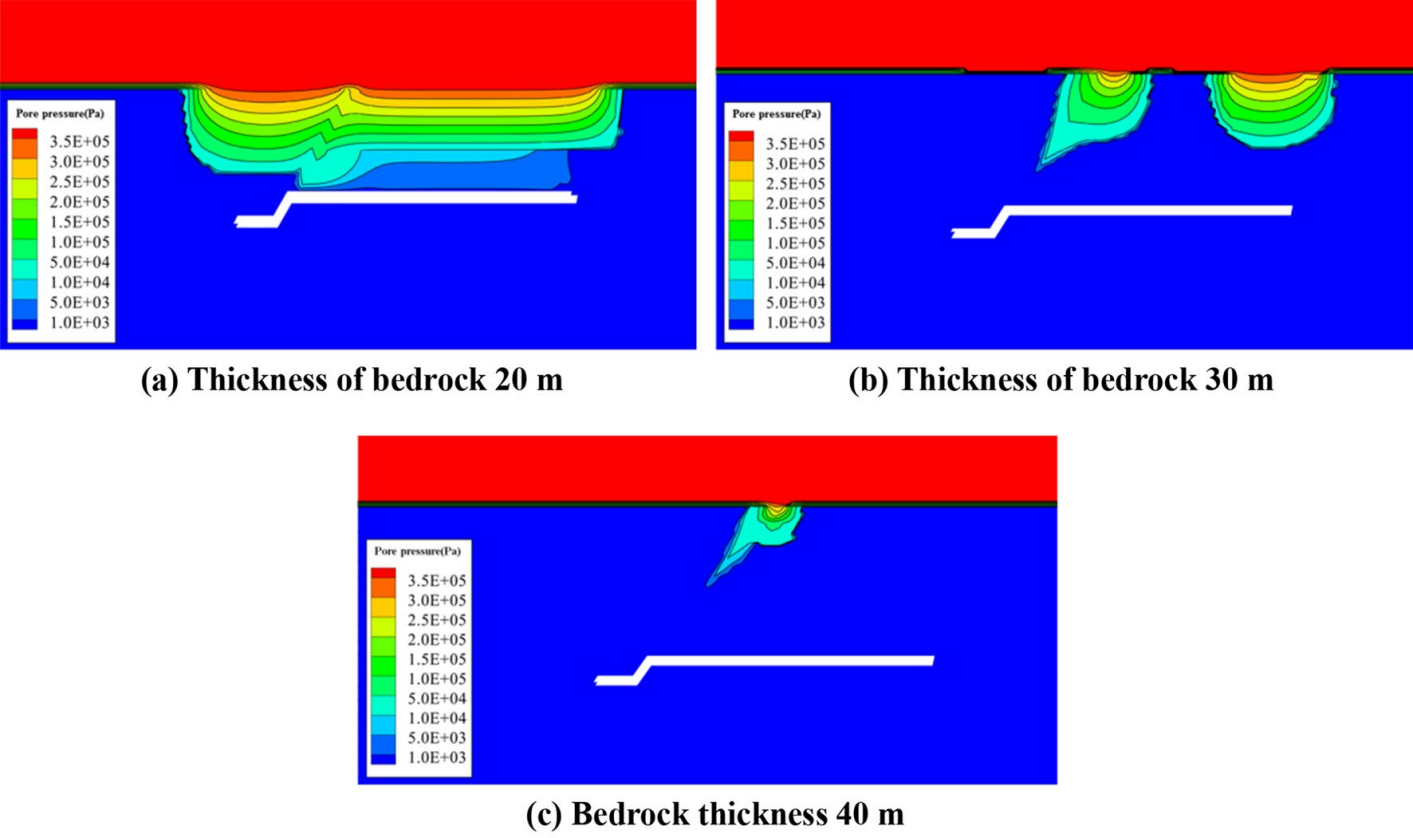
Model pore pressure diagram.

Figure 7 shows the distribution of model pore pressure at 32,000 steps when the working face passes through the fault 10 m for different bedrock thicknesses. For a bedrock thickness of 20 m, the overlying rocks and the upper clay layer are all destroyed, causing blocking of the resistance. The water capacity is largely reduced and the water in the loose layer seeps into the working face through the fault and the overlying fissure zone. Because the fault zone is more broken, the seepage speed of the water in the fault zone will be faster and it will merge into the working face earlier. Water inrush will occur along the entire surface. When the bedrock thickness is 30 m, the fractured rock mass above the fault zone and the open incision are the main seepage channel for water in the loose layer. For a bedrock thickness of 40 m, the bedrock thickness is larger than the development height of the fracture zone. The clay layer above the fault zone is damaged. Therefore, the fault zone is the main seepage channel.

This analysis indicates that when the working face advances the same distance, the lower the thickness of the bedrock, the lower the probability of fault failure. The water inrush mode of the working face varies with the different bedrock thicknesses. If all parts are destroyed, a serious risk of water inrush exist at various positions of the working face. When the bedrock thickness is 30 m, the fault zone and the cracks above the opening are the main water-conducting channels. For ae bedrock thickness of 40 m, water is mainly conducted by the fault zone. If the fault is reinforced in advance, the bond degree of the fault zone will be significantly strengthened. Therefore, the upper clay layer will not be affected by the fracture zone and no water conduction channel can form, realizing secure mining conditions across the fault under the thin bedrock.

## Simulation analysis of fault grouting reinforcement treatment

In order to realize cross-fault mining in the 331 working face and to prevent water gushing and sand inrush accidents of the working face caused by the activation of the fault, it is necessary to conduct grouting reinforcement on the Quince Tree No. 2 fault in the 331 working face. The grouting reinforcement can fill the interconnected surrounding rock cracks and the closed fissures that cannot be filled with grout can be closed and compressed^27^, thus improving the strength and water permeability of the rock. However, the effect of grouting reinforcement needs to be analyzed by numerical simulation. Considering the geological mining conditions of the Yangcun Coal Mine, we analyzed the grouting effect. The mechanical parameters selected for the grouting body are summarized in Table 2^28^.

**Table 2:** Mechanical parameters of fault zone after grouting.

Fault after grouting mechanical parameters	Bulk modulus (Gpa)	Shear modulus (Gpa)	Cohesion (MPa)	Friction Angle (°)	Tensile strength (MPa)	Permeability coefficient (cm/s)	Porosity (n)	Density (kg m^−3^)
Value	13.3	8.4	13.3	31	2.2	4.8e−7	0.15	2550

**Figure 8:**
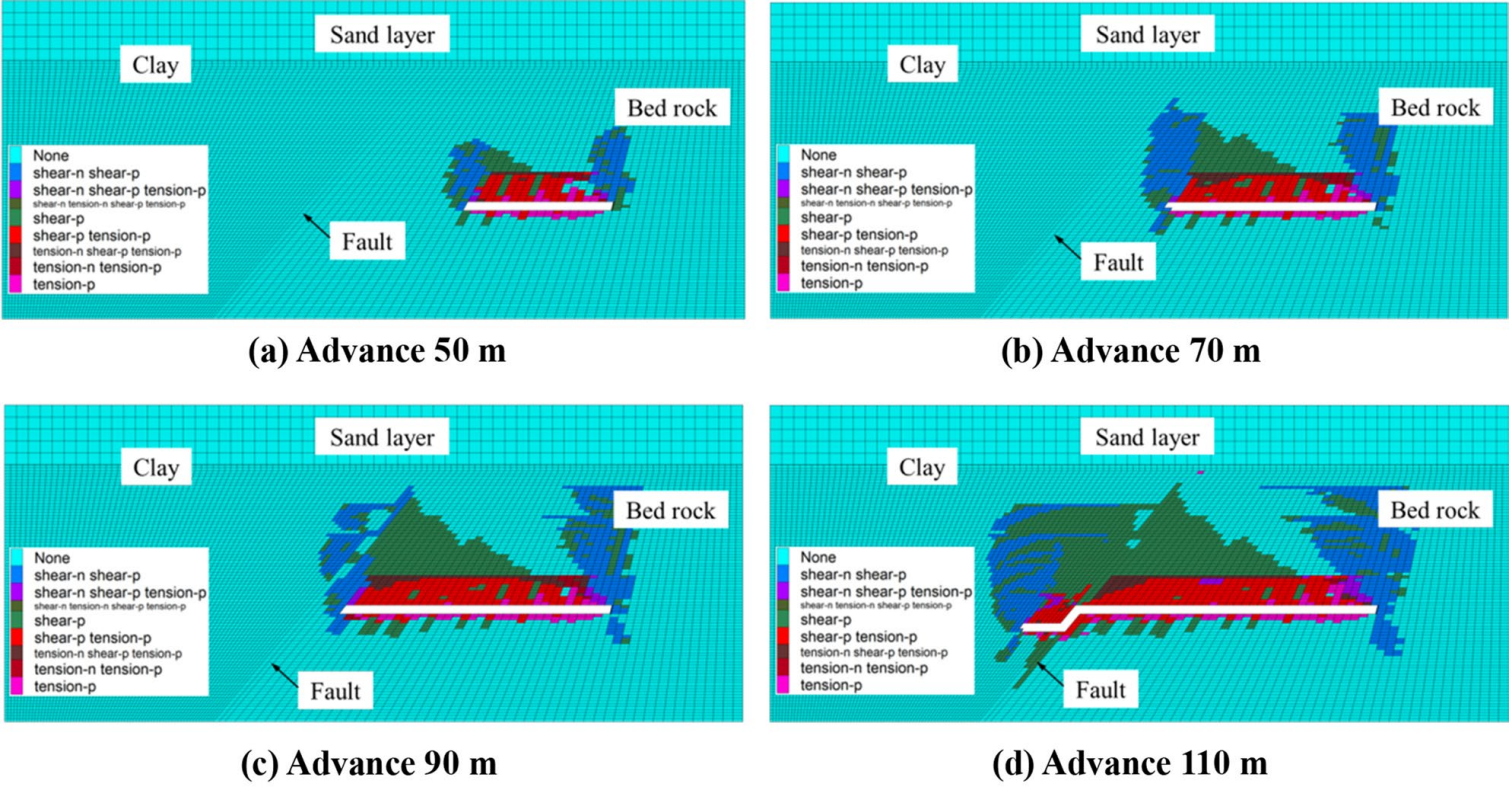
Plastic failure diagram of the model after grouting reinforcement.

Figure 8 shows the plastic failure diagram of the model after grouting reinforcement. When the working face is advanced to 70 m, the fault is not affect by premature failure. The failure height of the rock mass is the same, indicating that the fault has no additional influence on the fracture. When the working face passes through the fault zone for 10 m, the failure height of the fault zone and the height of the fracture zone reach a certain value and the clay layer is not affected by plastic damage, maintaining a good water resistance. The failure type of the fault zone is shear failure, indicating that the roof collapses normally without formation of a water conduction channel.

**Figure 9:**
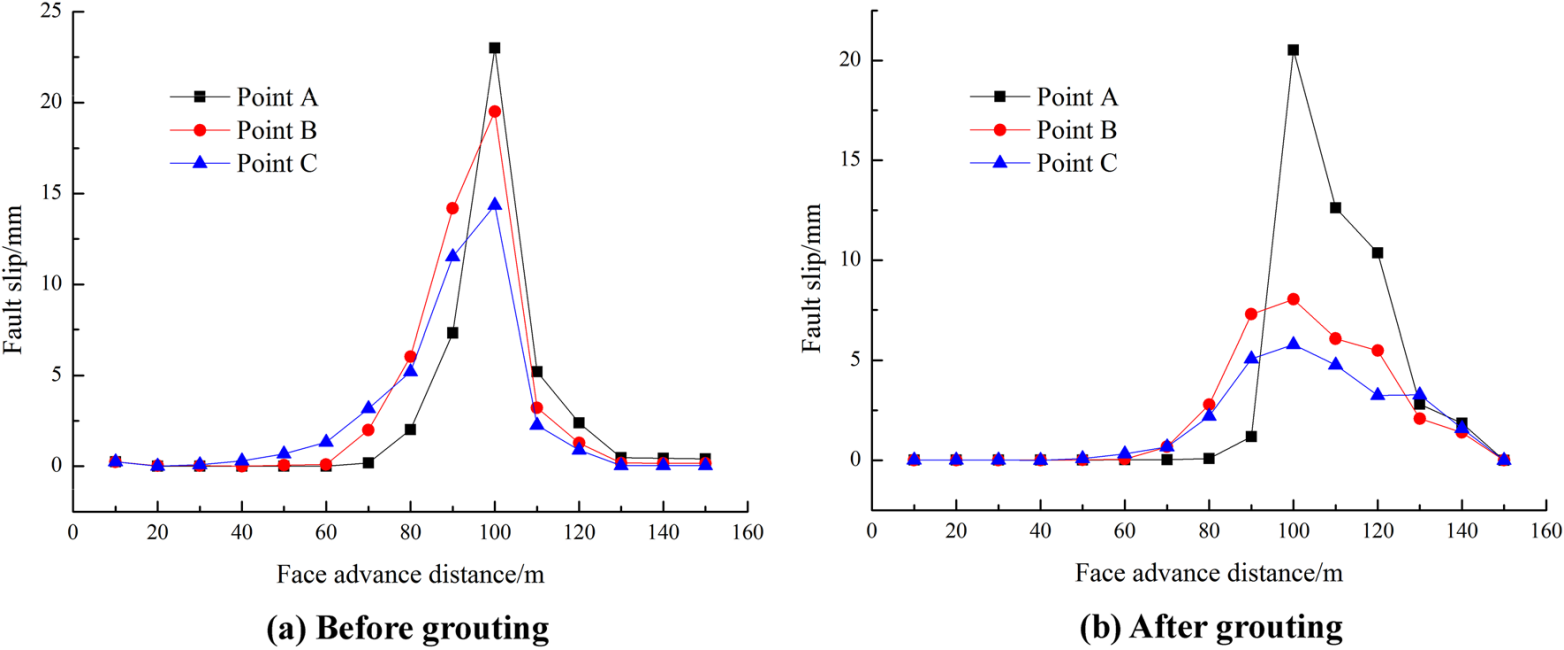
The amount of fault slip before and after grouting.

Figure 9 shows the slippage of the fault before and after grouting. The starting distance is used to describe the distance between the working face and the fault when the fault first responds. It can be seen from the figure that the starting distance of the fault at the high level is larger without grouting. The starting distance of the fault at the low level is the lowest and all reach the peak slip amount when the working face contacts the fault. The peak value of the slip amount of each layer is inversely proportional to the vertical distance from the fault. After the working face proceeded through the fault, the slip decreases gradually and tends towards a constant value after pushing 30 m through the fault. After the fault grouting, the starting distance of the fault and the peak value of the slip amount in each layer decrease and the peak values of the slip amount at monitoring points A, B, and C decrease accordingly by 10.8%, 58.8%, and 59.6%, indicating that after the fault is strengthened, the slip of the high-level fault is largely reduced. The displacement of monitoring point A is only moderately reduced due to its proximity to the caving zone.

To sum up, after fault grouting reinforcement, the fault zone will not be damaged in advance, the height of fracture development at the fault is suppressed, the slip of high-level faults is reduced, and the impact on the upper clay layer is low. Therefore, the formation of a water channel is prevented and safe recovery across fault is realized.

## Field practice verification analysis

**Figure 10:**
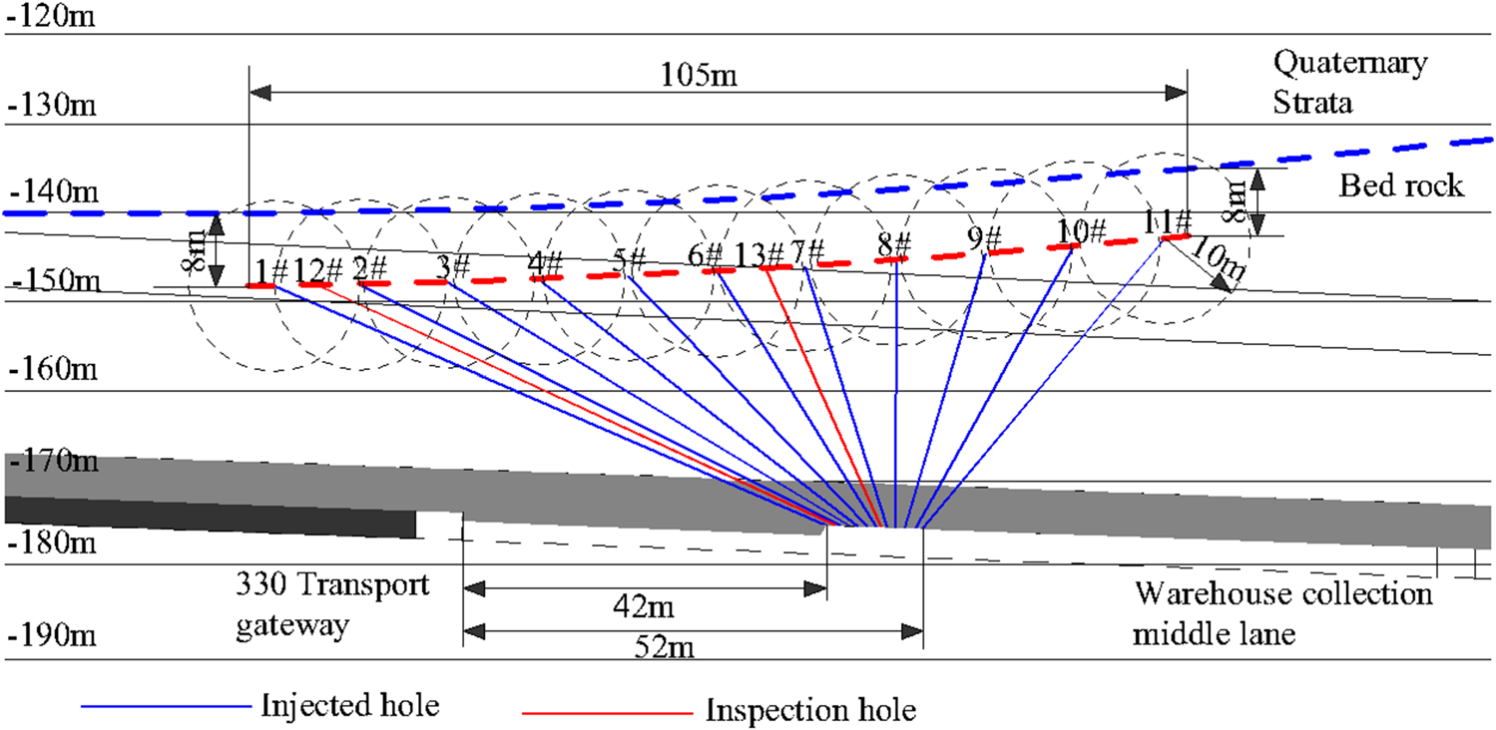
Plan of drilling holes for grouting reinforcement of Quince Tree No. 2 Fault.

To improve secure mining conditions, Yangcun Coal Mine carried out grouting reinforcement on the No. 2 fault in the 331 working face before mining, and then subsequently continued to mining production. The main purpose of this grouting was to strengthen the fault plane, delay the caving time of the roof near the fault, and reduce the development height of the caving zone due to the influence of the fault. The grouting hole is designed at the position of the fault plane 8 m below the Quaternary bottom boundary (Fig. 10), the grout splits and spreads in the fault zone to compact and reinforce the fault fracture zone. Part of the grout continues to diffuse upwards, enters the bottom containment, and reinforces part of the bottom sand layer.

The grouting body is made of pure cement slurry, mainly pure cement slurry and supplemented by double-liquid slurry. A cement with the grade of 42.5 is selected and the concentration of water glass is > 36 Baume degrees. It is suitable for filling wide cracks and cavities. Six different slurry ratios were prepared before grouting. According to the fluidity, rapid setting, economic analysis and actual situation of the slurry, the ratio in Table 3 was selected.

**Table 3:** Single liquid cement slurry preparation table.

Water-cement ratio	Cement dosage (kg/bag) PO42.5	Water (L)	Slurry volume (m^3^)
2:1	450/9	900	1.050

**Figure 11:**
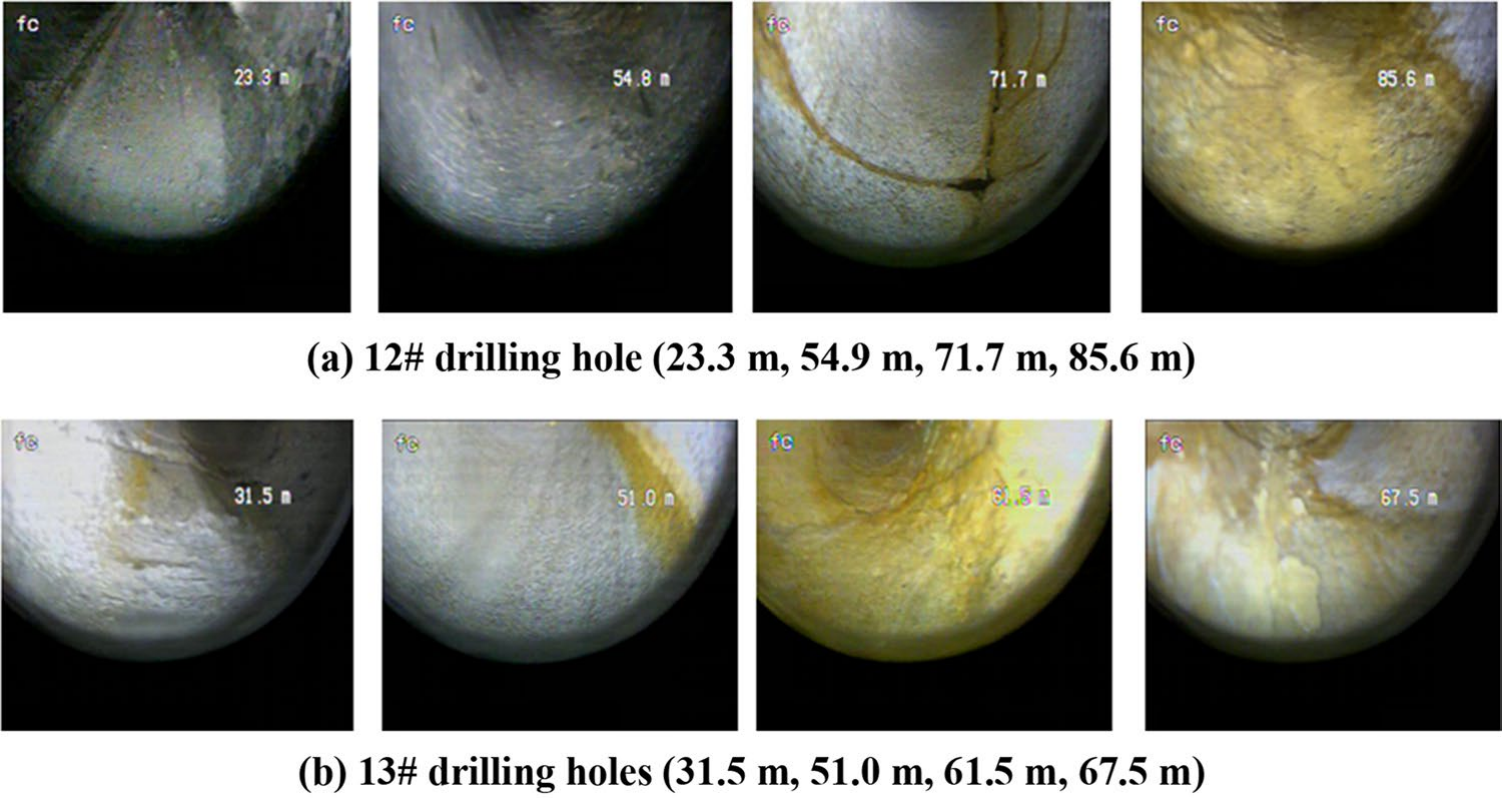
Inspection hole peep view.

11 preset grouting holes were present. The drilling interval is 10 m and two inspection holes are used for peeping and coring to check the grouting effect (Fig. 11). Only the holes 4# and 7# of the 13 drilling holes have water output. The water output is 0.1 and 2.0 m^3^/h, respectively. According to the analysis of the water output depth, the water output location should occur in the fault fracture zone. The overall water output holes are rare and single hole output. The water volume is low and there is no obvious water pressure in the effluent, indicating that the fault zone is weak in water richness and poor in water conductivity.

After grouting, we used two inspection holes to conduct drilling peeping (Fig. 11). The peeping depths of the 12# and 13# inspection holes are 86 m and 70 m, respectively, and the depths of the faults are 85.6 m and 61.5 m. The overall structure of the hole wall is relatively stable. Striking weathering fractures in the strata occur at the depth of 71 m and 50 m (Fig. 11). Fault fracture zones are missing in the peeping section. Combined with the grouting amount, drilling construction and coring comprehensive analysis, the conclusion is drawn: The filling is relatively dense and obvious water outflow phenomena are lacking, indicating a good effect of the grouting.

No water gushing, sand gushing, and roof pumping occurred in the 331 working face during the actual mining process, especially when the working face approached and pushed over the Queshu No. 2 fault, the roof fell normally, and abnormal situations did not occur. which verifies the accuracy of our numerical simulation analysis of grouting reinforcement. The safety stoping of the 331 working face was completed on September 5, 2021.

## Conclusions

1. The mechanical properties of the rock mass near the fault interface change and they are related to the cohesive force at the interface. The lower the required strength, on the contrary, the lower the cohesive force of the contact surface, the less strengthened the fault zone, and the larger the strength required for the rock at the contact surface.
2. The damage degree of the fault zone is related to the bedrock thicknesses during mining: The thinner the bedrock thickness, the lower the fault damage degree, for the same advancing distance. Different modes of water inrush occur: For a bedrock thickness of 20 m, the working face is fully inrush, for a thickness of 30 m the water inrush mainly depends on the fault zone and the fissure zone above the cut hole, and for a thickness of 40 m the water mainly follows the fault zone.
3. After fault grouting reinforcement, the fault zone of the working face did not fail in advance during the advancing process and had no effect on the development height of the fracture zone. It is small, effectively prevents the seepage of water in the loose layer and provides a meaningful scientific basis for cross-fault mining of coal mine working face under similar conditions.

## Data Availability

The primary data used to support the findings of this study are available from the corresponding author upon request.
